# The Rice G Protein γ Subunit *DEP1/qPE9–1* Positively Regulates Grain-Filling Process by Increasing Auxin and Cytokinin Content in Rice Grains

**DOI:** 10.1186/s12284-019-0344-4

**Published:** 2019-12-16

**Authors:** Dongping Zhang, Minyan Zhang, Yong Zhou, Yuzhu Wang, Jinyu Shen, Hongyingxue Chen, Lin Zhang, Bing Lü, Guohua Liang, Jiansheng Liang

**Affiliations:** 1 Department of Biology, Southern University of Science and Technology, Shenzhen, 518055 China; 2 Jiangsu Key Laboratory of Crop Genetics and Physiology/Co-Innovation Center for Modern Production Technology of Grain Crops, Key Laboratory of Plant Functional Genomics of the Ministry of Education, Yangzhou University, Yangzhou, 225009 China

**Keywords:** G-protein, *DEP1/qPE9–1*, Starch biosynthesis, Grain filling, Hormones, *Oryza sativa*

## Abstract

Heterotrimeric G protein-mediated signal transduction is one of the most important and highly conserved signaling pathways in eukaryotes, which involves in the regulation of many important biological processes. As compared with those in mammals and *Arabidopsis thaliana*, the functions of rice heterotrimeric G protein and their molecular mechanisms are largely unknown. The rice genome contains a single G_α_ (*RGA1*) and G_β_ (*RGB1*), and five G_γ_ (*RGG1*, *RGG2*, *GS3*, *DEP1/qPE9–*1, and *GGC2*) subunits. Recent genetic studies have shown that *DEP1/qPE9–1*, an atypical putative G_γ_ protein, is responsible for the grain size as well as the dense and erect panicles, but the biochemical and molecular mechanisms underlying the control of grain size are not well understood. Here, we report that rice plants carrying *DEP1/qPE9–1* have more endosperm cells per grain than plants contain the *dep1/qpe9–1* allele. The *DEP1/qPE9–1* line has a higher rate and more prolonged period of starch accumulation than the *dep1/qpe9–1* line. Additionally, the expression of several essential genes encoding enzymes catalyzing sucrose metabolism and starch biosynthesis is higher in the *DEP1/qPE9–1* line than in the *dep1/qpe9–1* line, especially from the mid to late grain-filling stage. Grains of the *DEP1/qPE9–1* line also have higher contents of three phytohormones, ABA, auxin and cytokinin. Exogenous application of auxin or cytokinin enhanced the starch accumulation and the expression of genes encoding grain-filling-related enzymes in the grains of *dep1/qpe9–1*, whereas ABA produced no effects. Based on these results, we conclude that *DEP1/qPE9–1* positively regulates starch accumulation primarily through auxin and cytokinin, which enhance the expression of genes encoding starch biosynthesis during the mid to late grain-filling stage, resulting in increased duration of the grain-filling process.

## Background

Rice grain yield is a complex trait that is influenced by both the grain sink capacity and grain sink activity/grain filling efficiency (i.e., photo-assimilate inter-conversion and starch accumulation). The former is controlled by the numbers of spikelet per panicle and the number and size of endosperm cells per spikelet/grain, both of which determine the final grain number per panicle and the grain size, whereas grain filling efficiency is closely related to the grain filling rate and the duration of grain filling.

Rice grain size, not like the wheat grains, is physically limited by the spikelet hull. However, the final grain size is mainly determined by the number and size of endosperm cells per grain, which is positively associated with grain weight. Therefore, grain size is an important agronomic trait for yield improvement in rice. The developmental process of grain is the processes of cell proliferation and cell expansion, as well as the processes of photoassimilate interconversion and starch accumulation. Recently, a series of studies are enhancing our understanding on the molecular events related to developmental regulation of grain size and shape (reviewed by Li et al. 2018; Li et al. 2019a). Almost all of the identified quantitative trait loci (QTLs)/genes so far are involved in regulating grain size by modulating the number of cells through stimulating cell division and/or cell elongation. However, few QTLs/genes have been reported to regulate the division and expansion of the endosperm cells and the grain filling processes.

The heterotrimeric G protein (hereafter G protein)-mediated signal transduction pathway is considered one of the essential signaling mechanisms and regulates various critical physiological and molecular processes in both mammals and higher plants (Urano and Alan 2014). In this signaling pathway, the G protein, which is well-known to consist of three different subunits (α, β, and γ), acts as a signal intermediator in the transduction of numerous external signals (Milligan and Kostenis 2006). *Arabidopsis* G-protein Gamma subunit 3 (*AGG3*), which represents a novel class of canonical γ subunits in *Arabidopsis*, is widely spread throughout the plant kingdom but is not present in animals (Urano et al. 2013). Recently, *AGG3* has been proposed to be an important regulator of organ size and a mediator of stress responses in *Arabidopsis*. Roy Choudhury et al. (2013) overexpressed *AGG3* in *Camelina* and found that it increased the seed size, seed mass and seed number per plant by 15%–40%, effectively resulting in a significantly higher oil yield per plant. In addition, *AGG3* has also been shown to affect the guard cell K^+^ channel activity, morphological development, ABA responses and cell proliferation (Chakravorty et al. 2011; Li et al. 2012; Roy Choudhury et al. 2013). These observations draw a secure link between the roles of *AGG3* in regulating two critical yield parameters (seed traits and plant stress responses) and reveal an effective biotechnological tool to increase agricultural crop yield dramatically.

Homologs of *AGG3* in rice have been identified as major quantitative trait loci (QTL) for grain size and yield. The major rice QTLs, i.e., *GRAIN SIZE 3* (*GS3*) and *DENSE AND ERECT PANICLE1* /*PANICLE ERECTNESS* (*DEP1/qPE9–1*)] have 29.4% and 22.5% amino acid sequence identities, respectively, with *AGG3* (Li et al. 2012). As an atypical G_γ_ protein, rice *GS3* has been proposed to affect cell proliferation negatively, whereas *DEP1/qPE9–1* plays a positive role (Fan et al. 2006; Huang et al. 2009; Zhou et al. 2009). These findings suggest that *AGG3* and its homologs in rice may have divergent functions.

In present study, we used a stable transgenic line, WYJ8 (*DEP1/qPE9–1*), and its non-transgenic counterpart WYJ8, which carried the *dep1/qpe9–1* allele, to investigate how *DEP1/qPE9–1* control the development of endosperm cells and grain filling process. Our results had clearly showed that *DEP1/qPE9–1* significantly stimulates starch accumulation and grain filling by enhancing cell division and the activities of enzymes catalyzing photo-assimilate inter-convention and starch biosynthesis. Auxin and cytokinin might act as intermediate regulators to regulate grain filling process.

## Methods

### Plant Material and Treatments

The experiment was carried out at the farm of Yangzhou University (32°30′N, 119°25′E) during the rice (*Oryza sativa*) growing season (from early May to early September). A stable *DEP1/qPE9–1* transgenic rice line and the donor Wuyunjing 8 (*dep1/qpe9–1*) were grown in the field (see: Zhou et al. 2009). At the heading stage, 500 uniformly growing and headed panicles (1–2 panicles per plant) were chosen, and spikelets on the selected panicles with the same flowering date were labeled for each cultivar/line. The flowering date and position of each spikelet on the labeled panicles were recorded. Approximately 45 labeled panicles were sampled at each time point from flowering to maturity. Half of the sampled grains were frozen in liquid nitrogen for at least 2 min and then stored at − 80 °C for subsequent analyses. The other half of the grains were dried at 80 °C for approximately 72 h to a constant weight and used for the starch analyses.

The hormone treatment consisted of 80 plastic pots with planted rice (three hills per pot) maintained under open field conditions. Each pot (0.6 m in height with 0.5 m and 0.3 m top and bottom diameters, respectively) was filled with sandy loam soil that contained the same nutrient contents as the field soil. The sowing date and cultivation were the same as those for the field experiment. After flowering, 50 μM ABA, 10 μM NAA or 10 μM 6-BA was sprayed at a rate of 10 ml per pot on the top of the plants (spikes) every 3 days. The hormones were applied between 16:00 h and 18:00 h. All of the solutions contained ethanol and Tween 20 at final concentrations of 0.1% (v/v) and 0.01% (v/v), respectively. The control plants were sprayed with the same volume of deionized water containing the same ethanol and Tween 20 concentrations. Each treatment consisted of 13 pots, and the labeled spikelets were sampled.

### Isolation and Counting of Endosperm Cells

The endosperm cells of the grains were isolated and counted according to the procedures described by Singh and Jenner (1982). Briefly, fixed grains (10 grains from five labeled panicles) were transferred to 0.7:1 (v:v) ethanol:water and dehulled. The dehulled grains were transferred to 0.5:1 (v:v) ethanol, 0.25:1 (v:v) ethanol and finally to distilled water for 5~7 h prior to dissection of the endosperms. The endosperms were isolated under a dissecting microscope and dyed using Delafield’s hematoxylin solution for 24~30 h, washed several times with distilled water and then hydrolyzed in a 0.1% cellulase solution at 40 °C. The degree of recovery of the cells after digestion was 80~95%. The isolated endosperm cells were diluted to 2~10 ml according to the developmental stage of the endosperm, from which 5 subsamples (20 ml per subsample) were transferred to a counting chamber (1-cm^2^ area). The endosperm cell number in 10 grids per counting chamber was counted using a light microscope. The number of nuclei was counted as the number of endosperm cells. The total endosperm cell numbers were calculated using the following equation (Liang et al. 2001):

$$ \mathrm{Endosperm}\ \mathrm{cell}\ \mathrm{number}=\frac{\frac{\frac{\mathrm{cell}\ \mathrm{number}}{\mathrm{grid}}\times \mathrm{counting}\ \mathrm{chamber}\ \mathrm{area}}{\mathrm{area}\ \mathrm{of}\ \mathrm{each}\ \mathrm{grid}}\times \mathrm{total}\ \mathrm{volume}\ \mathrm{of}\ \mathrm{sample}}{\mathrm{volume}\ \mathrm{of}\ \mathrm{each}\ \mathrm{subsample}} $$

### Gene Expression Analysis

Total RNA was extracted from the grains (10~20 grains from five labeled panicles) using the RNAprep Pure Plant Kit (cat. no. DP441; Tiangen, Beijing, China). The HiScript II Q Select RT SuperMix (Vazyme, Nanjing, China) was used for cDNA synthesis. The transcript level of each gene was measured by qRT-PCR using the 7500 Real-Time PCR System (ABI) with the PowerUp™ SYBR® Green Master Mix (Thermo Fisher Scientific, San Jose, USA). Gene expression was quantified during the logarithmic phase using expression of the housekeeping gene *Ubq* (LOC_Os03g13170) as an internal control. Three biological replicates were performed for each experiment. The primer sequences used for qRT-PCR are described by Wang et al. (2013).

### Hormone Quantification

The IAA, ABA and tZR levels were determined by Zoonbio Biotechnology Co., Ltd. (Nanjing, China). Approximately 0.5 g of the samples were ground in a precooled mortar that contained 5 ml of extraction buffer composed of isopropanol/hydrochloric acid. The extract was shaken at 4 °C for 30 min. Then, 10 ml of dichloromethane was added, and the sample was shaken at 4 °C for 30 min and centrifuged at 13,000 rpm for 5 min at the same temperature. We extracted the lower organic phase. The organic phase was dried under N_2_, dissolved in 150 μl of methanol (0.1% methane acid) and filtered with a 0.22-μm filter membrane. The purified product was subjected to high-performance liquid chromatography-tandem mass spectrometry (HPLC-MS/MS) analysis. The HPLC analysis was performed using a ZORBAX SB-C18 (Agilent Technologies) column (2.1 mm × 150 mm; 3.5 mm). The mobile phase A solvent consisted of methanol/0.1% methanoic acid, and the mobile phase B solvent consisted of ultrapure water/0.1% methanoic acid. The injection volume was 2 μl. The MS conditions were as follows: the spray voltage was 4500 V; the pressure of the air curtain, nebulizer and aux gas were 15, 65 and 70 psi, respectively; and the atomizing temperature was 400 °C.

### Enzyme Activity Assays

The dehulled grains (10 grains from five labeled panicles) were homogenized with a prechilled mortar and pestle in 100 mM HEPES buffer (pH 7.5) containing 8 mM MgCl_2_, 2 mM EDTA, 50 mM 2-mercaptoethanol, 12% (v/v) glycerol and 1% (w/v) polyvinylpyrrolidone (PVP). After centrifugation at 30,000 x g for 10 min at 4 °C, the supernatant was desalted through a dialytic membrane. The dialysis buffer contained 5 mM HEPES-NaOH, pH 7.4, 5 mM MgCl_2_, 1 mM EDTA and 0.5 mM DTT. The enzyme activity of SUS (in the cleavage direction), SS and BE was determined as described by Nakamura et al. (1989), Jiang et al. (2003) and Tang et al. (2009). The grain starch contents were determined according to Lü et al. (2008).

### Statistical Analysis

The data are presented as the mean ± SD. The SPSS 16.0 software was used for all statistical analyses. Statistical significance was determined for independent biological samples using Student’s t-test for comparison of two groups and one-way ANOVA for comparison of three or more groups. Differences were considered statistically significant when *P* < 0.05. An asterisk (*) is presented when *P* ˂ 0.05.

## Results

### *DEP1/qPE9–1* Positively Controls Grain Size and Grain Weight

**Fig. 1:**
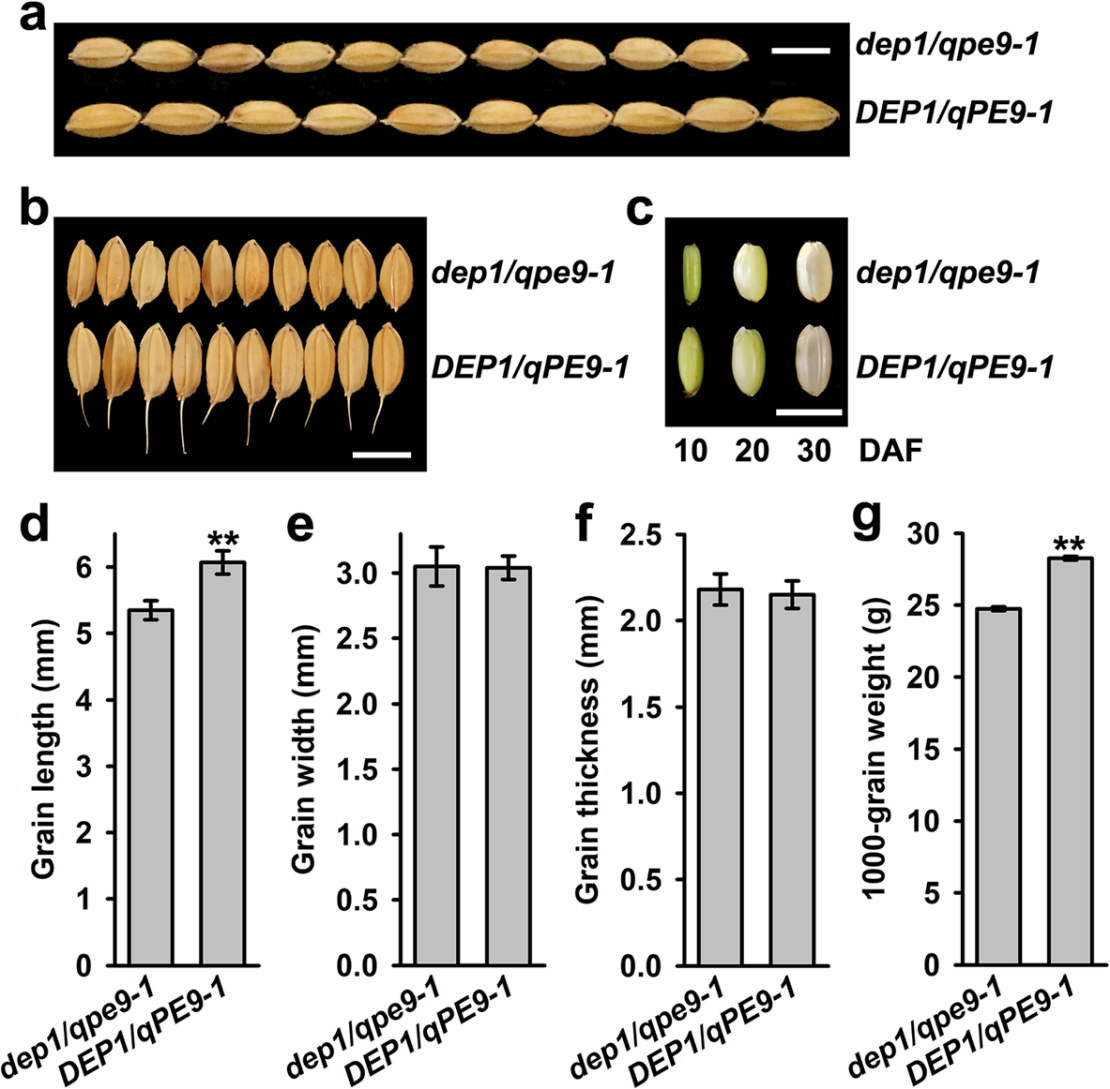
Grain performance of *DEP1/qPE9–1* and *dep1/qpe9–1*. **a** Comparisons of grain length between *DEP1/qPE9–1* and *dep1/qpe9–1*. Scale bar: 0.5 cm. **b** Comparisons of grain width between *DEP1/qPE9–1* and *dep1/qpe9–1*. Scale bar: 0.5 cm. **c** Brown rice during grain development. Scale bar: 0.5 cm. **d-g** Grain length, grain width (*n* = 30 grains from 5 panicles) and 1000-grain weight (*n* = 4 from 4 panicles) of *DEP1/qPE9–1* and *dep1/qpe9–1.* Data are presented as the mean ± SD. * *P* < 0.05, ** *P* < 0.01

Using QTL analysis, two independent research groups originally identified rice *DEP1/qPE9–1* as controlling the panicle morphology and grain number per panicle (Huang et al. 2009; Zhou et al. 2009). The previous study also showed that *DEP1/qPE9–1* regulates grain size and grain weight (Sun et al. 2018; Li et al. 2019b). However, the biochemical and molecular mechanisms underlying the control of the rice grain size and weight are largely unknown. In the present study, we firstly compared the differences in grain size of Wuyunjing 8, which contains the *dep1/qpe9–1* allele (hereafter *dep1*/*qpe9–1*), and the *DEP1/qPE9–1* transgenic line (hereafter *DEP1/qPE9–1*). The results showed that the final grain length of *dep1*/*qpe9–1* was approximately 12% shorter than that of *DEP1/qPE9–1* (Fig. 1a, d), but no significant differences in the final grain width and thickness were observed between *dep1*/*qpe9–1* and *DEP1/qPE9–1* (Fig. 1b, e and f). The grain size difference of two genotypes was much more significant during the early stage of grain filling (Fig. 1c). As a consequence, the final 1000-grain weight of *DEP1/qPE9–1* was remarkably higher than that of *dep1/qpe9–1* (Fig. 1g).

### *DEP1/qPE9–1* Controls Caryopsis Size Mainly through Enhancing Endosperm Cell Proliferation but Not the Cell Size

**Fig. 2:**
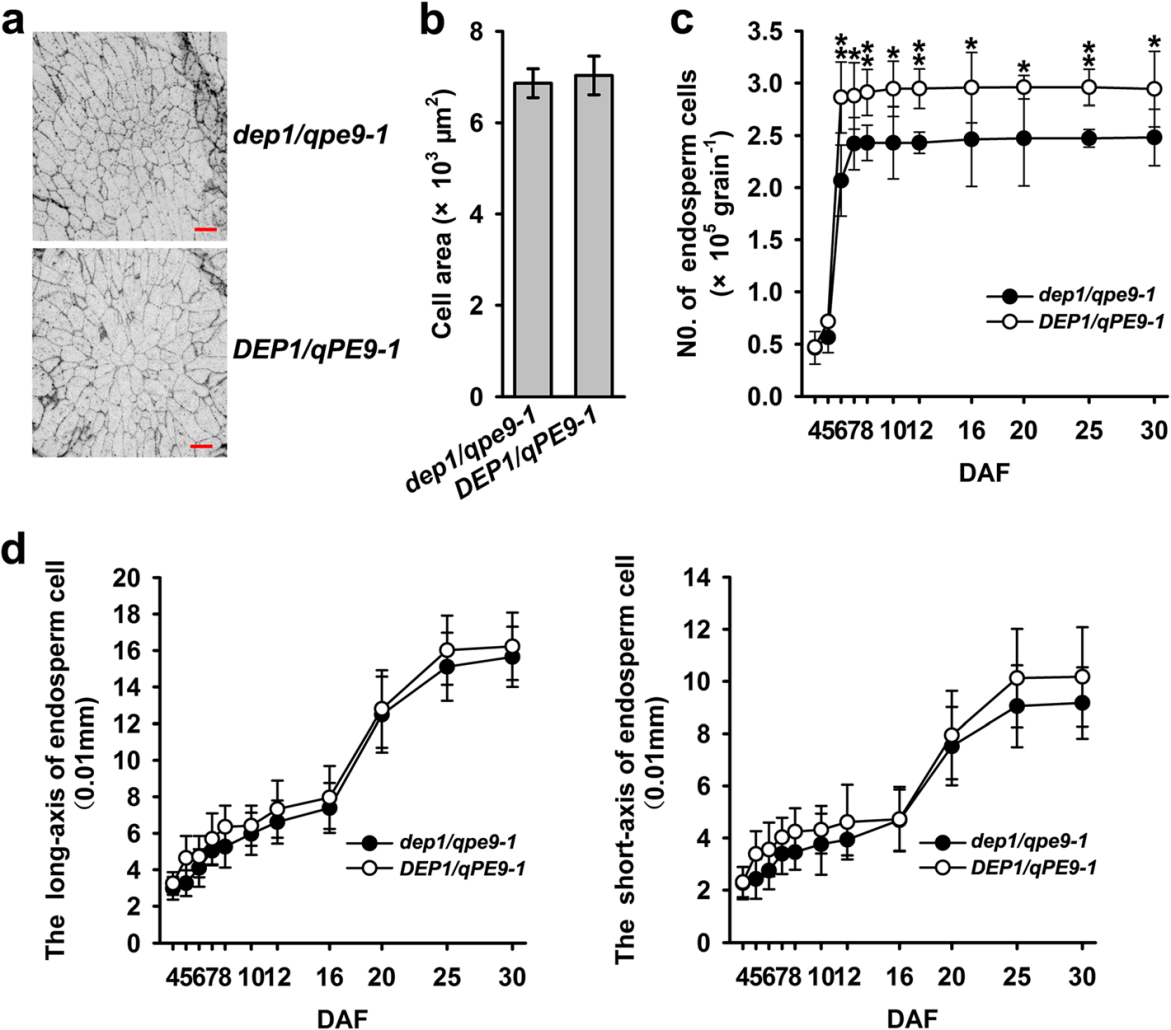
Histological analyses of endosperms at maturity and changes in endosperm size after fertilization in *DEP1/qPE9–1* and *dep1/qpe9–1*. **a** Cross-sections of the endosperm between the dorsal and central point showing the cell sizes and numbers. Scale bars: 100 μm. **b** Comparison of cell numbers in the endosperm cross-sections (*n* = 5 endosperms from 5 panicles). **c** Changes in the numbers of endosperm cells during grain filling (*n* = 10 endosperms from 5 panicles). **d** The long and short axes of endosperm cells during grain development (*n* = 20 endosperm cells from 5 grains). Data are presented as the mean ± SD. * *P* < 0.05, ** *P* < 0.01

Grain size and shape are determined by cell proliferation and size of either the hull and the endosperms (caryopsis) (Orozco-Arroyo et al. 2015). The hull size determines the final size of the grain, but the final hull size was determined immediately after heading and when the hull size is determined, the final grain size and grain weight are determined both by the number and size of the endosperm cells and by grain filling processes. Here, we compared the number and area of endosperm cells in cross-sections of mature grains from *dep1*/*qpe9–1* and *DEP1/qPE9–1*. No significant difference in the endosperm cell area was observed between the *dep1*/*qpe9–1* and *DEP1/qPE9–1* line (Fig. 2a, b), and the long and short axes of the *dep1/qpe9–1* endosperm cells were slightly shorter than those of *DEP1/qPE9–1*, but the differences were not significant throughout the whole grain filling stage (Fig. 2d). These results suggested that the number, but not the size, of the endosperm cells, might be responsible for the difference of the grain size. We further analyzed the dynamic changes of the numbers of endosperm cells of the two genotypes during grain filling stage, and the results showed that the number of endosperm cells per grain increased rapidly during early grain filling stage and that the peak values were reached at the 9th day after flowering (9 DAF). The numbers of endosperm cell per grain of *DEP1/qPE9–1* was significantly higher than that of *dep1/qpe9–1* (Fig. 2c). These results suggest that *DEP1/qPE9–1* control grain size mainly through enhancing the endosperm cell proliferation, especially during the early stage of grain development.

### *DEP1/qPE9–1* Increases Starch Content through Prolonging the Duration of the Grain-Filling Process

**Fig. 3:**
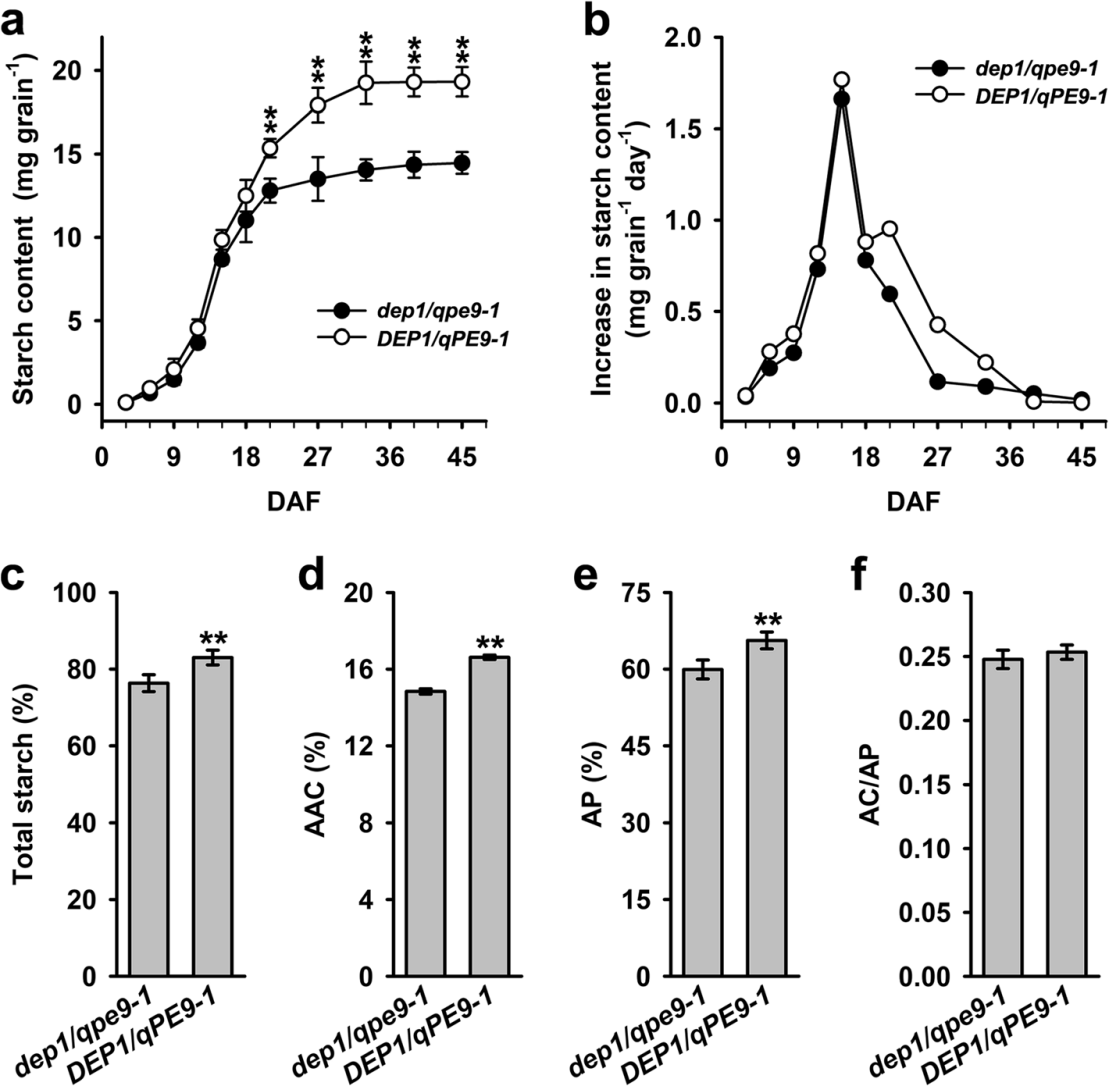
Starch accumulation during grain filling. **a** Starch accumulation of grains during the grain-filling stages (*n* = 5). **b** The rate of starch accumulation during grain filling. **c-e** The content of total starch, amylose (AC) and amylopectin (AP) in the flour (*n* = 5). **f** The amylose/amylopectin ratio. Data are presented as the mean ± SD. * *P* < 0.05, ** *P* < 0.01

Grain weight is controlled by both the grain size (i.e., the size and the number of the endosperm cells) and the grain filling processes (i.e. sugar inter-conversion and starch biosynthesis). In this experiment, we also compared the differences of two genotypes in starch accumulation during grain filling processes. The starch content of the grains increased rapidly after flowering in both genotypes (Fig. 3a). However, the starch content of the *DEP1/qPE9–1* grains was much higher than those of the *dep1/qpe9–1*, and the final starch content of the *DEP1/qPE9–1* grain was approximately 10% higher than that of *dep1/qpe9–1* (Fig. 3a, c). During the early stage of grain filling (i.e., before 18 DAF), no significant difference in starch accumulation was detected between two genotypes. However, the *DEP1/qPE9–1* grains continued to accumulate starch till 33 DAF rapidly, and then kept a relatively stable state till the end of grain filling, whereas, the maximum starch content was reached at 27 DAF in *dep1/qpe9–1* grains, and remained relatively stable until the end of grain filling (Fig. 3a). The rate of starch accumulation increased rapidly after flowering, reached its maximal level at 15 DAF and then decreased as the grain-filling process continued. Again, the difference in the rate of starch accumulation of two genotypes during grain filling was observed mainly at the mid to late stage of grain filling (after 18 DAF) (Fig. 3b). These data clearly indicate that *DEP1/qPE9–1* positively controls the starch accumulation process during grain filling and prolongs the duration of the grain-filling process. These results, in combining with the above findings, also implied that the differences in weight between two genotypes are not only resulting from the grain size, but also from grain filling processes. Interestingly, mutation of *DEP1/qPE9–1* did not cause alteration in the amylose: amylopectin ratio (Fig. 3f), because contents of amylose (AC) and amylopectin (AP) were both higher in *DEP1/qPE9–1* compared with *dep1/qpe9–1* (Fig. 3d and e).

### *DEP1/qPE9–1* Stimulates Starch Biosynthesis through Enhancing the Expression of Genes Encoding Starch Biosynthesis-Related Enzymes and their Activity in Rice Grains

**Fig. 4:**
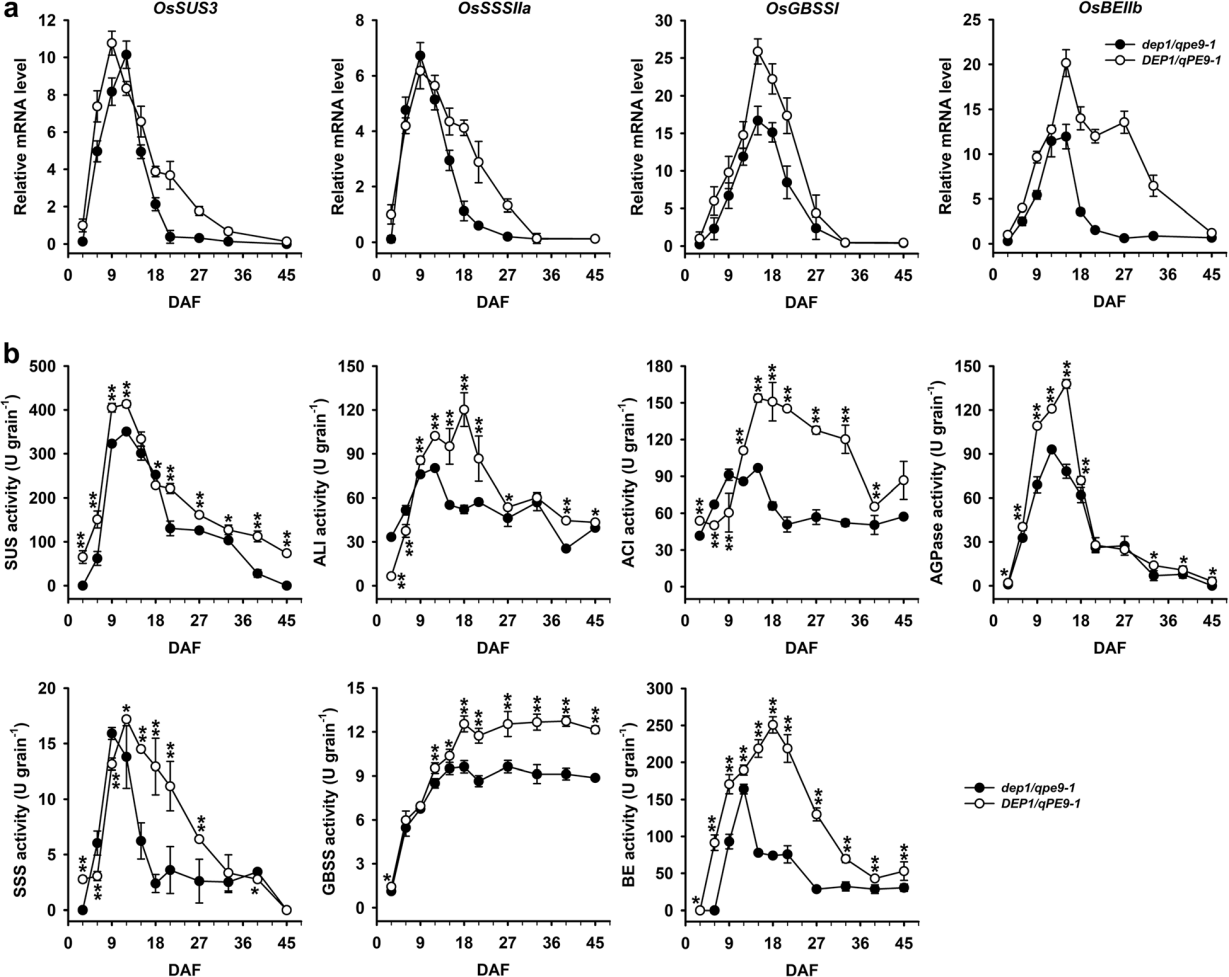
Expression of several starch biosynthesis genes and changes in the activities of these enzymes during grain filling. **a** *OsSUS3*, *OsSSIIa*, *OsBEIIb* and *OsGBSSI* expression levels during grain filling (*n* = 3); **b** Changes in the enzyme activities during grain filling (*n* = 3), 1 U = 1 μg/min/mg protein. Data are presented as the mean ± SD. * *P* < 0.05, ** *P* < 0.01

Grain filling processes are in fact a series of reactions including sugar inter-conversion and starch biosynthesis and are highly regulated by both genetic and environmental factors. Many enzymes involve to catalyze these reactions, including sucrose synthase (SUS), invertase (INV), ADP-glucose pyrophosphorylase (AGPase), soluble sucrose synthase (SSS), granule bound starch synthase (GBSS), branching enzyme (BE) and debranching enzyme (DBE), etc., and all of them are well-known to play key roles in the regulation of sucrose degradation, ADP-glucose and starch biosynthesis (Liang et al. 2001; Lü et al. 2008; Tang et al. 2009; also see reviews by Tetlow et al. 2004; Keeling and Myers 2010; Zeeman et al. 2010). In order to elucidate the molecular mechanisms underlying the regulation of grain filling processes, we examined the expressional patterns of these genes during grain-filling stage. The results showed that the transcript levels of *OsSUS3*, *OsSSSIIa, OsGBSSI* and *OsBEIIb* were significantly higher in the grains of *DEP1/qPE9–1* than that of *dep1/qpe9–1*, especially at the mid to later stages of grain-filling (Fig. 4a), and no significant differences were detected for other genes (results not shown). We also measured the activities of enzymes encoded by these genes of the two genotypes during grain filling. As shown in Fig. 4b, a significant difference in AGPase activity between two genotypes occurred at the rapid grain filling stages, that is, from 5 to 18 days after flowering and for other five enzymes, i.e., SUS, invertase (ALI and ACI), SSS, GBSS and BE, *DEP1/qPE9–1* grains had higher enzyme activities than *dep1/qpe9–1* during almost whole grain filling stages, but the most differences were observed at the mid to late stages of grain filling. Taken together, our results suggest that *DEP1/qPE9–1* plays key roles in controlling grain filling processes through its effects on the expression of several key genes and the activities of the encoded enzymes related to sucrose metabolism and starch biosynthesis during grain filling.

### *DEP1/qPE9–1* Changes the Homeostasis of Hormones in the Endosperm Cells

**Fig. 5:**
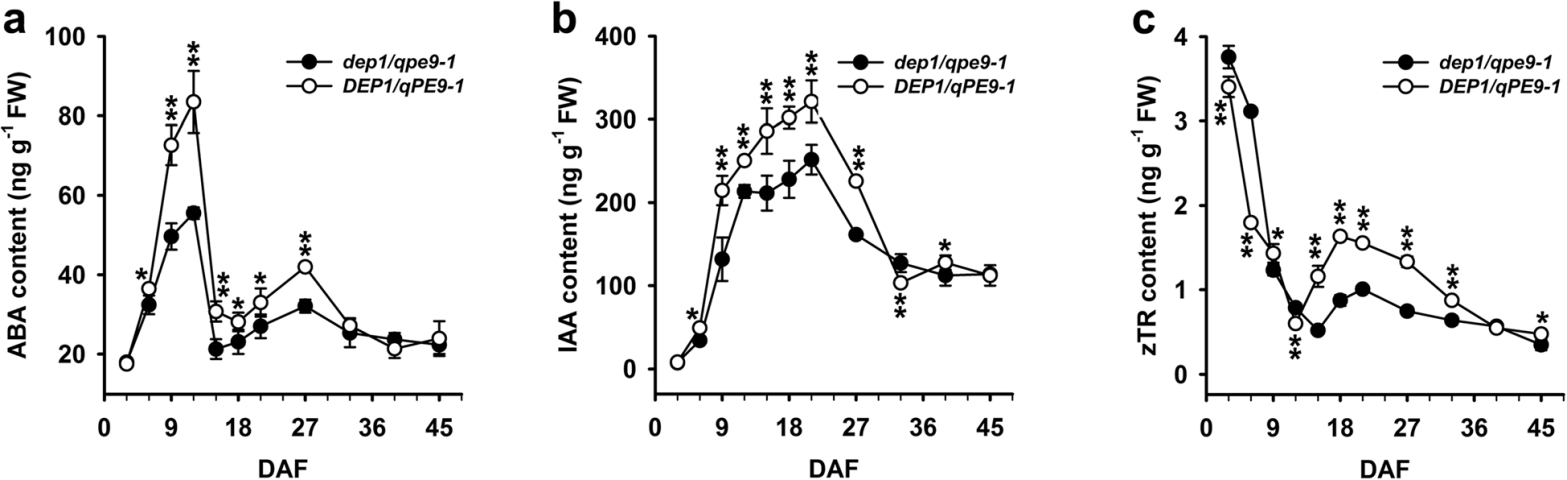
Changes in the hormone contents during grain filling. **a** Changes in the ABA level in rice endosperm cells during seed development (*n* = 3). **b** Changes in the IAA level in rice endosperm cells during seed development (*n* = 3). **c** Changes in the tZR level in rice endosperm cells during seed development (*n* = 3). Data are presented as the mean ± SD. * *P* < 0.05, ** *P* < 0.01

Lots of evidence have shown that grain filling is also a highly regulated process and it has many reports that plant hormones play important roles in regulating the grain filling (Tang et al. 2009; Zhu et al. 2011; Zhang et al. 2016). We were interested in investigating whether *DEP1/qPE9–1* controlled the grain-filling process also through its effects on plant hormone homeostasis. Significant changes in the endogenous ABA, auxin (IAA) and cytokinin (tZR) contents in the grains were found during grain filling (Fig. 5). The ABA content increased during the early grain filling stage until 12 DAF and then decreased until the end of grain filling. The endogenous ABA content in the *DEP1/qPE9–1* grains was always higher than that in *dep1/qpe9–1* (Fig. 5a). Very similar patterns of dynamic changes in the IAA content were observed, with the *DEP1/qPE9–1* grains having higher IAA concentration than *dep1*/*qpe9–1* during the grain-filling stages (Fig. 5b). High contents of tZR were detected at the beginning of grain filling followed by a rapid decrease to a relatively low and stable level at mid to late stage of grain filling. Compared with *dep1/qpe9–1*, rice plants carrying *DEP1/qPE9–1* produced less tZR at the early early stage of grain development. However, much higher contents of tZR were observed in *DEP1/qPE9–1* grains than that in *dep1/qpe9–1* grains during mid to late stage of grain development (Fig. 5c).

### Application of Hormones Partially Rescued the Starch Content of *dep1/qpe9–1*

**Fig. 6:**
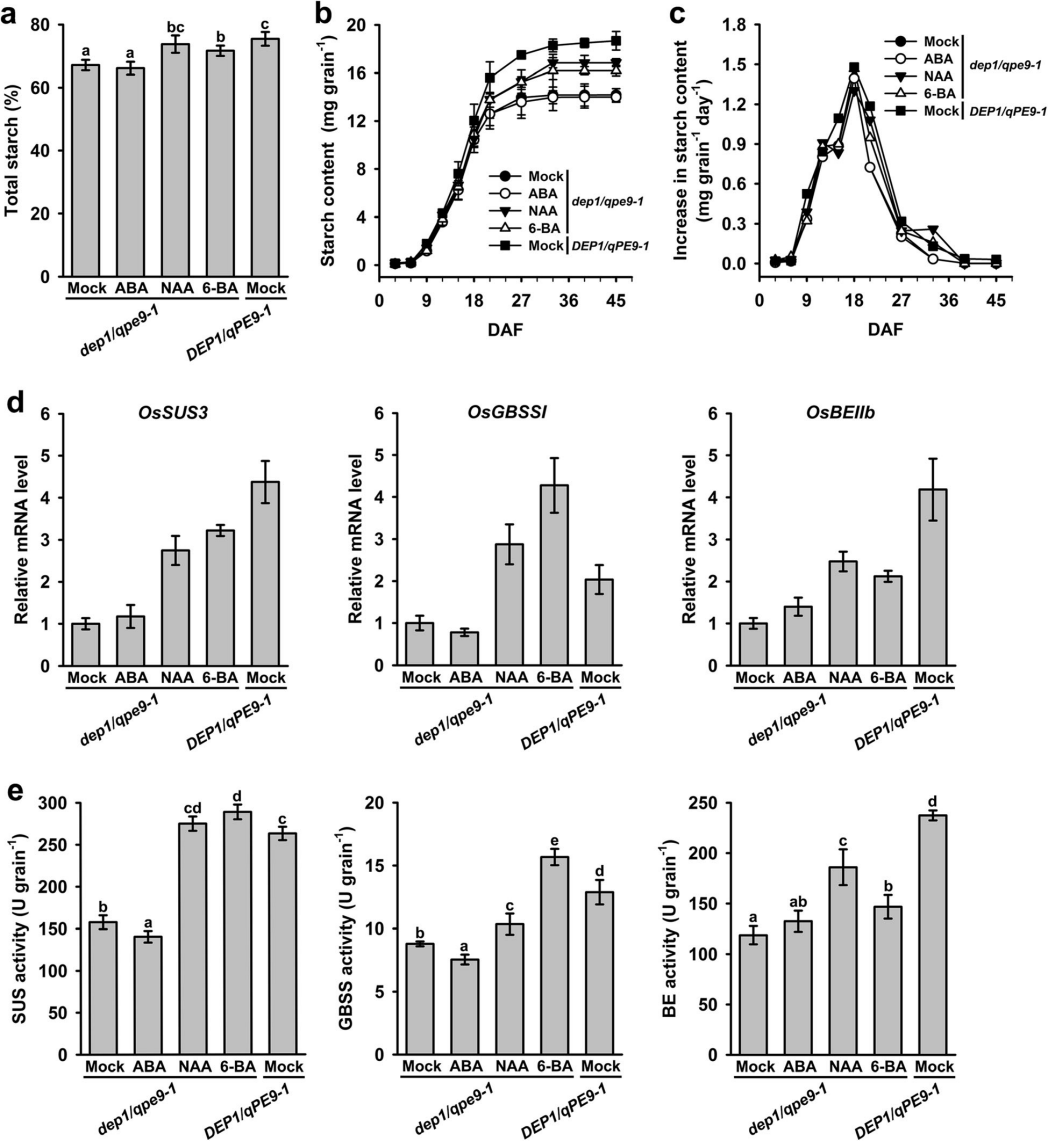
Effects of exogenous ABA and IAA applications on starch biosynthesis. **a** Effects of exogenous ABA, NAA and 6-BA on starch accumulation (*n* = 5). **b** Effects of exogenous hormones on starch accumulation of grains during the grain-filling stages (*n* = 5). **c** Effects of exogenous hormones on the rate of starch accumulation during grain filling. **d** Effects of exogenous hormones on *OsSUS3*, *OsBEIIb* and *OsGBSSI* expression at 20 DAF (*n* = 3). **e** Effects of exogenous hormones on SUS, BE and GBSS activities at 20 DAF (*n* = 3), 1 U = 1 μg/min/mg protein. Data are presented as the mean ± SD. * *P* < 0.05, ** *P* < 0.01

To verify the relationships between *DEP1/qPE9–1* and the plant hormones during the grain-filling stages, exogenous hormones were applied to the developing grains during filling stage, and starch accumulation was analyzed. The panicles of 20 DAF were sprayed with ABA, 6-BA (6-Benzylaminopurine) for the cytokinin treatment and NAA (1-naphthaleneacetic acid) for auxin treatment. As shown in Fig. 6a, ABA treatment had no effects on starch accumulation. However, starch content in grains of *dep1/qpe9–1* was enhanced and reached the level of *DEP1/qPE9–1* by NAA and 6-BA applied. Moreover, application of 6-BA, but not NAA, stimulated the endosperm cell proliferation in *dep1/qpe9–1* line, but not in *DEP1/qPE9–1* plant (data not shown). These results implied that NAA and 6-BA can partially restore the starch content of *dep1/qpe9–1* grains to that of the *DEP1/qPE9–1*, and the difference of starch accumulation between two genotypes was, at least in part, due to these two hormones. Furthermore, grain-filling dynamic analysis showed that both NAA and 6-BA prolonged grain filling period, and finally increase starch synthesis (Fig. 6b, c). Application of NAA and 6-BA also stimulated the expression of *OsSUS3*, *OsBEIIb* and *OsGBSSI* genes and the activities of their encoded enzymes in *dep1/qpe9–1* (Fig. 6d, e). These results in combination with above results indicate the lower grain starch content and weight in *dep1/qpe9–1* line is, to some extent, due to the lower content of auxin and cytokinin during grain filling period, especially from mid to late stage of grain filling.

## Discussion

The rice *DEP1/qPE9–1* gene was formerly identified by two independent research groups as a gene that controls panicle erectness, grain number per panicle and, consequently, grain yield (Huang et al. 2009; Zhou et al. 2009). Sequential alignment at the amino acid level showed that *DEP1/qPE9–1* was a homolog of *AGG3*, which is a γ subunit of the *Arabidopsis* heterotrimeric G protein, although its similarity and degree of homology were not so high. AGG3 is a recently discovered novel protein that is almost twice as large as a typical G_γ_ protein. This protein exhibits a high degree of similarity with canonical G_γ_ proteins at the N-terminus, whereas the C-terminus is plant-specific and contains an extremely high number of cysteine residues (Chakravorty et al. 2011; Botella 2012). In plants, the G_γ_ and G_β_ subunits are tightly bound and exert their effects as a dimer in both the active and inactive states of the heterotrimeric G protein. Recent studies showed that, based on the interaction between DEP1/qPE9–1 and RGB1 (G_β_ subunit of rice), DEP1/qPE9–1 is a G_γ_ protein in rice (Botella 2012; Sun et al. 2014).

The functions and molecular mechanisms of DEP1/qPE9–1 as a new G_γ_ subunit in rice are largely unknown. Because *DEP1/qPE9–1* has been cloned and identified as a gene controlling the panicle size and rice shape (Huang et al. 2009; Zhou et al. 2009), it is reasonable to assume that *DEP1/qPE9–1* may play important roles in regulating grain development and the grain-filling process. Rice grain formation and grain filling are rather complicated processes that involve approximately 21,000 genes, including 269 genes that are closely related to various physiological and biochemical pathways (Zhu et al. 2003). Proteomic research indicates that 123 proteins and 43 phosphoproteins are involved in the regulation of the grain-filling process (Zhang et al. 2014). Furthermore, the grain-filling process is also regulated by several kinds of small chemical molecules, such as plant hormones (e.g., ABA, IAA, cytokinin and ethylene), which fluctuate considerably during the grain-filling period (Yang et al. 2001, 2003, 2006; Tang et al. 2009). Although the biochemical pathway of carbohydrate inter-conversion occurred in endosperm cells has clearly been elucidated during grain filling, the biochemical and molecular mechanisms controlling rice grain size and weight are remained to be explored.

Overexpression of *Arabidopsis* atypical G_γ_ (*AGG3*) promotes seed and organ growth by increasing cell proliferation, and *AGG3-*mutated lines have a small seed size (Chakravorty et al. 2011; Li et al. 2012; Roy Choudhury et al. 2013). Similar results have been reported for rice plants. *GS3* and *DEP1/qPE9–1* are involved in controlling either the grain size or the panicle size in rice (Fan et al. 2006; Huang et al. 2009; Zhou et al. 2009). However, in contrast to *Arabidopsis*, where *GS3* negatively regulates the grain size, *DEP1/qPE9–1* positively regulates both the grain and panicle size (Fan et al. 2006; Huang et al. 2009; Zhou et al. 2009). Why this considerable difference exists between these plant species remains unclear. Recently, Sun et al. (2018) proposed a model that reduces grain size by blocking the interaction of RGB1 with other G_γ_ proteins, such as DEP1 and GGC2.

A comparison of the panicle morphology, grain size and final grain weight clearly indicated that *dep1/qpe9–1* had a smaller size and lower grain weight than *DEP1/qPE9–1*, implying that *DEP1/qPE9–1* positively regulated the grain size and final grain weight (Fig. 1). Our previous results and other groups revealed that the grain size and final grain weight were largely determined by two factors/processes: the number and size of endosperm cells (i.e., the sink size) and the grain-filling process, including the grain-filling rate and duration (i.e., the sink activity) (Liang et al. 2001). The smaller grain size and lower final grain weight of *dep1/qpe9–1* are the result of fewer endosperm cells and lower starch accumulation after flowering, the latter is partly due to reducing the duration of the grain-filling process (Figs. 2 and 3). However, the mechanisms underlying how *DEP1/qPE9–1* controls endosperm cell proliferation and starch accumulation are unknown.

More than ten proteins/enzymes are directly involved in the biochemical pathways of carbohydrate interconversion and starch biosynthesis during rice grain filling (Tetlow et al. 2004; Ohdan et al. 2005; Zhu et al. 2011). What is the underlying cause of lower starch accumulation in *dep1/qpe9–1* aside from the lower numbers of endosperm cells, as compared with those of *DEP1/qPE9–1*? In other words, do significant differences exist in the expression of key protein/enzyme-encoding genes or the activity of these key enzymes between the two genotypes? To answer these questions, we compared differences in the expression of genes encoding several key enzymes that catalyzed carbohydrate interconversion and starch biosynthesis between the two genotypes used in this experiment. Our results clearly indicate that the lower expression levels of several key genes that encode enzymes catalyzing starch biosynthesis and the lower activities of these enzymes in the *dep1/qpe9–1* grains during the mid to late grain filling stage are the most important factors resulting in the lower grain weight (Fig. 4).

The grain-filling process is a highly regulated process that involves both genetic and environmental factors. Plant hormones play important roles in grain growth and development (Tang et al. 2009; Zhu et al. 2011; Zhang et al. 2016). However, little is known about the detailed mechanisms by which plant hormones regulate the grain-filling process. Yang et al. (2001) suggested that an altered hormonal balance, especially a decrease in GA and an increase in ABA, enhanced the remobilization of pre-stored carbohydrate in stem-sheath to the grains and accelerated the grain-filling rate. In addition, IAA treatment increased spikelet growth and development in distal branches (Patel and Mohapatra 1992). Recently, studies in peas provided direct evidence that auxin was required for a normal seed size and starch accumulation. The mutant of *TAR2*, which is an IAA biosynthesis gene, induces the formation of small seeds with reduced starch content and a wrinkled phenotype at the dry stage. Application of the synthetic auxin 2,4-D partially reversed the wrinkled phenotype but did not restore the starch content of the mutant seeds to that of the WT (McAdam et al. 2017). Our results showed considerable differences in the dynamics of the endogenous hormone (ABA, auxin and cytokinin) levels in the grains during grain filling. The levels of these endogenous plant hormones were significantly lower in the *dep1/qpe9–1* than in the *DEP1/qPE9–1* grains during the mid to late grain-filling stage (Fig. 5). Based on the effects of exogenous ABA, auxin and cytokinin on starch accumulation and the expression of genes that encode several key enzymes catalyzing starch biosynthesis, we conclude that both auxin and cytokinin increase the duration of the grain-filling process (Fig. 6). The positive control of *DEP1/qPE9–1* on the grain-filling process occurs largely through changing the biosynthesis of these plant hormones.

## Conclusions

In summary, rice grain filling is a very complicated process that involves photoassimilate (mainly sucrose) translocation from photosynthetic sources (i.e., leaves and leaf sheaths), sucrose degradation, transmembrane transport and starch synthesis in the grains (i.e., the sink) (Liang et al. 2001; Lü et al. 2008; Tang et al. 2009). More than ten enzymes/proteins have been reported to be involved in these biochemical processes (Tetlow et al. 2004; Ohdan et al. 2005; Zhu et al. 2003). Our results showed that the novel G protein γ subunit, *DEP1/qPE9–1,* increased the numbers of endosperm cells and positively regulated starch biosynthesis, which enhanced the grain size and weight. *DEP1/qPE9–1* also enhanced the accumulation of auxin and cytokinin. In turn, these hormones regulate the expression of genes encoding several key enzymes that catalyze starch biosynthesis during grain filling stage and consequently affect the final grain weight.

## Data Availability

All data supporting the conclusions of this article are provided within the article.

## Abbreviations

ABA: Abscisic acid
ACI: Acid invertase
AGPase: ADP-glucose pyrophosphorylase
ALI: Alkaline invertase
BE: Branching enzyme
DBE: Debranching enzyme
GBSS: Granule bound starch synthase
IAA: Indole-3-acetic acid
INV: Invertase
NAA: 1-naphthylacetic acid
SS: Starch synthases
SUS: Sucrose synthase
tZR: Trans-zeatin riboside
